# Evaluation of the effectiveness of phonological awareness education developed for children aged 48–60 months

**DOI:** 10.3389/fpsyg.2026.1940818

**Published:** 2026-10-15

**Authors:** Leyla Şahin, Umut Akcil, Filiz Erbay

**Affiliations:** 1 Near East University, Faculty of Education, Nicosia, Türkiye; 2 School of Health Sciences, KTO Karatay Universitesi, Konya, Türkiye

**Keywords:** action research, early childhood, early literacy, phonological awareness, preschool education

## Abstract

Phonological awareness skills developed in early childhood are accepted as one of the strongest predictors of children’s future reading success. However, there are only limited practical studies on how these skills can be supported in a systematic and sustainable manner in preschool classroom settings. This study aims to design and implement developmentally structured activities to support preschool children’s phonological awareness skills and to systematically examine the implementation process and changes observed among the participating children within an action research framework. The research was conducted using an action research design, and 14 children who were 48–60 months old and 30 preschool teachers participated in the study. During the data collection process, teacher interviews, observations, researcher logs, and the Early Literacy Test were employed. Prior to the intervention, it was found that the phonological awareness mean scores of children were low (mean = 9.85). After the eight-week implementation period, the mean pre-test score was 9.85, whereas the mean post-test score was 25.78. The highest post-test mean scores were observed for syllable blending and initial sound matching skills. The findings showed higher post-test phonological awareness scores than pre-test scores among the participating children. The study addresses an important gap in the literature by providing a practical example of phonological awareness instruction in an early childhood classroom setting.

## Introduction

Linguistic and cognitive skills acquired during early childhood are accepted among the strongest developmental indicators which determine the future academic success of children (Cabell et al., 2021; Snow and Matthews, 2016). Particularly findings indicating that the foundations of reading skill begin to be shaped well before formal education process have significantly increased the international interest in the field of early literacy (Quinn et al., 2021). It is reported that today millions of children start elementary school without developing adequate early literacy skills, which is related to literacy difficulties, low academic performance, and educational inequalities in following years (UNESCO, 2023). For this reason, early childhood education is not a preparation phase which supports only the social–emotional development of children; it is also evaluated as a critical development phase where the cognitive, linguistic and phonological foundations of academic literacy are structured.

Phonological awareness, which is one of the basic components of early literacy, is defined as the children’s ability to consciously pay attention to the phonetic structure of spoken language, to distinguish sounds, and to process these sound structures (Gillon, 2002). This structure, consisting of sub-skills such as rhyme awareness, syllable separation, sound blending, recognition of initial and final phonemes, and phoneme manipulation plays a central role in establishing the relationship between written and spoken language among children (Piasta et al., 2020). Recent longitudinal studies display that phonological awareness skills developed in preschool period demonstrate a strong correlation with reading accuracy, fluency, and reading-comprehension performance during elementary school years (Quinn et al., 2021; Cabell et al., 2021). It is emphasized that especially from the perspective of learning letter-sound matching and development of decoding skills, phonological awareness is a critical prerequisite skill. In this context, it is reported that children who do not experience adequate levels of phonological awareness in preschool period have higher probability of suffering from difficulties in formal reading instruction process.

However, despite the existence of theoretical and empirical findings regarding the importance of phonological awareness in literature, the question of how these skills can be systematically and sustainably supported in preschool classroom settings is still being addressed only to a limited extent. A significant body of current research demonstrates that phonological awareness is a variable which predicts future reading success; however, it provides only limited explanation to developmentally sequenced intervention models which can be applied by teachers and integrated to natural classroom routines (Veríssimo et al., 2021; Choi et al., 2024). Especially multi-dimensional studies which address the initial performance of children, teacher requirements, intervention process and application results in combination are very limited, which implies that there is a gap between theoretical knowledge and classroom applications. Recent debates on early childhood emphasize that it is necessary to go beyond studies which only measure children’s performances and to develop intervention models with high applicability in the classroom (Justice et al., 2017; Cabell et al., 2021).

It is also reported that although preschool teachers are key implementers in supporting the early literacy skills, most teachers lack adequate systematic guidance regarding the developmental sequence in which phonological awareness skills should be supported, what teaching strategies will be used, and how these skills will be integrated into daily classroom routines (Veríssimo et al., 2021). Studies show that in the absence of structured teaching processes which provide review opportunities, children’s phonological awareness development are left solely to natural language interaction (Carson et al., 2013). However, it is reported that planned and developmentally structured interventions presented in early childhood period significantly support children’s early literacy development (Piasta et al., 2020). For this reason, supporting phonological awareness skills through direct instruction is seen as crucial not only for language development, but also for reducing early academic inequalities.

A review of studies on Turkish-speaking children reveals that phonological awareness studies mostly focus on describing the existing skill levels of children (Turan and Akoğlu, 2011). On the contrary, studies that address the teacher experiences, classroom application processes and structured intervention effects in natural preschool settings are rather limited (Veríssimo et al., 2021; Choi et al., 2024). Especially the scarcity of application-based studies that examine the impact of developmentally sequenced activities on the phonological awareness performance of children is remarkable (Piasta et al., 2020). This situation leads to the applied empirical evidence regarding the early literacy development of Turkish-speaking children remaining limited in the international literature. Therefore, studies on the applicable, teacher-supported, and developmentally structured intervention models in early childhood classrooms are needed.

Based on this necessity, the current study aimed to design, implement, and evaluate the effectiveness of developmentally sequenced learning activities intended to support phonological awareness skills in preschool children. Within the scope of this study, the opinions of preschool teachers regarding phonological awareness skills were examined first, and then the current skill levels of children were determined. A structured intervention program was developed based on these initial findings and the impact of application process on children’s phonological awareness performance was evaluated. In addition, through observations conducted throughout the research process, the study sought to provide qualitative evidence regarding the applicability of the developed activities in natural classroom settings. To this end, the study aims to contribute to filling an important gap in the literature by offering a practical and multidimensional intervention model for teaching phonological awareness in early childhood education. From this viewpoint, the basic question of the study was defined as “What should learning activities be like to develop phonological awareness skills in the preschool period?” and the study addressed the following research questions:

1. What are the preschool teachers’ perspectives on phonological awareness skills in the preschool period?
2. What are the baseline phonological awareness skill levels of preschool children aged 48–60 months?
3. What kind of action plan should be developed to improve phonological awareness skills?
4. What are the phonological awareness skill levels of children prior to and following the action research implementation?
5. What are the researcher’s observations regarding the implementation of activities designed to target phonological awareness skills?”

## Method

### Research design

An action research design was employed in this study in line with the specified objectives. Action research is a research approach that aims to systematically address problems arising from practice in educational settings by planning, implementing and evaluating solutions to this problem, and improving the process continuously (Kemmis et al., 2014; Creswell and Creswell, 2023). From this perspective, action research offers the researcher the opportunity to assume the roles of both practitioner and observer, while directly contributing to the development of educational practices.

By nature, action research studies consist of multiple stages exhibiting a cyclical structure. In the literature this process is known as the “action research cycle” wherein the researcher undergoes multiple stages throughout the research process (Stringer, 2020). This study adopted a seven-stage action research process proposed by Stringer (2020). The overall application stages of the study are presented in Figure 1.

**Figure 1:**
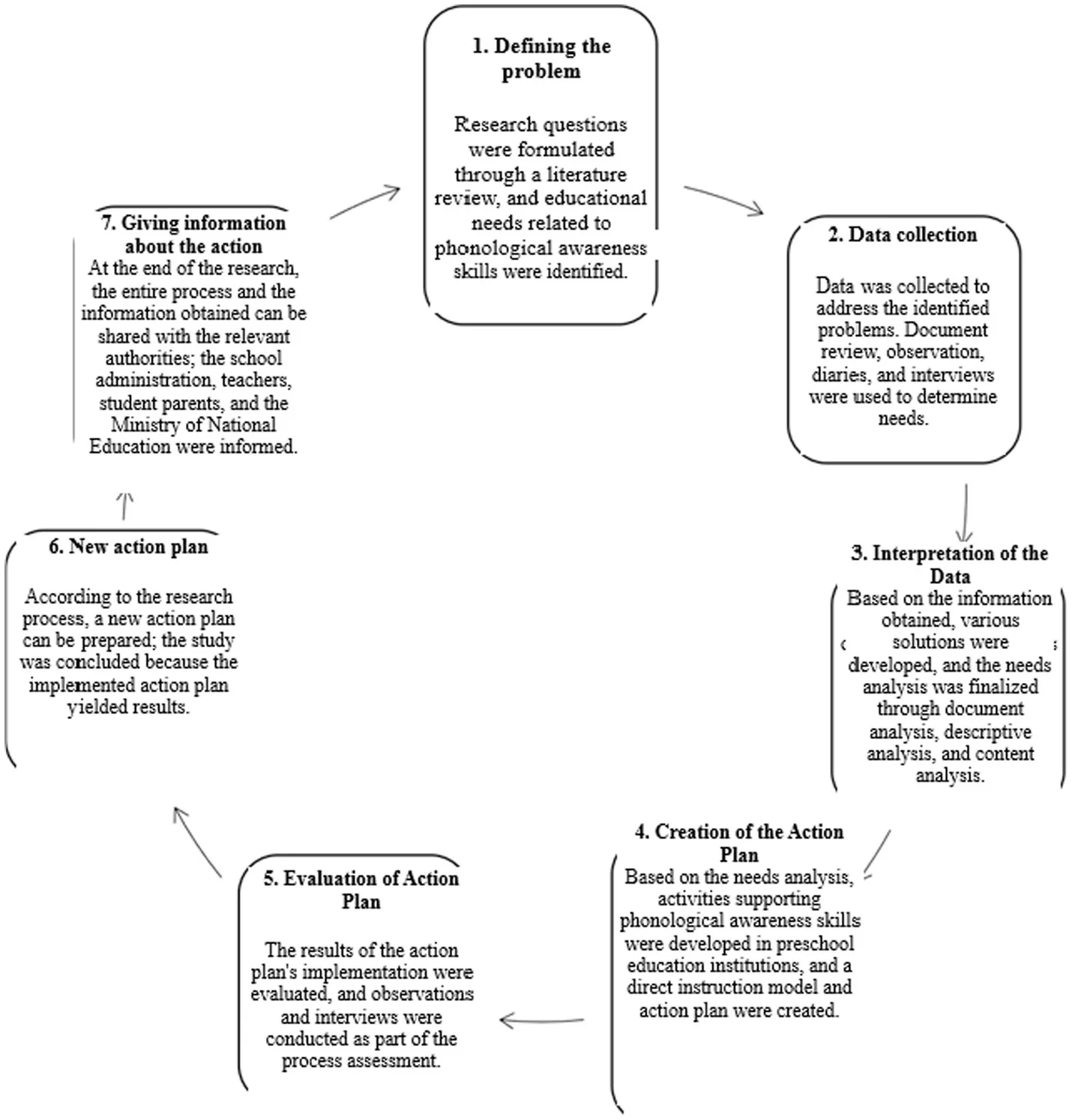
General application stages of action research process used in the study.

The mentioned seven stages in this study were realized as follows: at the problem definition stage, literature review and classroom observations were used to determine the educational needs of children regarding phonological awareness skills. At the data collection stage, document analysis, observation, interviews and researcher–practitioner journals were used to comprehensively examine the determined needs and research questions. At the data interpretation stage, document, descriptive, content and needs analysis were conducted based on the acquired data. At the action plan creation stage, activities were performed to support the phonological awareness skills of pre-school children in line with the identified educational needs and an implementation plan was developed in line with a suitable instructional model. At the action plan evaluation stage, the participation and reactions of children and the difficulties they experienced at the application stage were observed, and data were obtained through researcher–practitioner journals and interviews. At the new action plan stage, it was assessed whether there was a need to revise the action plan implemented based on the evaluation results. Observations from the initial implementation indicated that the overall action plan was appropriate for the intended teaching process. Therefore, no substantial revision of the overall action plan was considered necessary. Finally, at the action reporting stage, the findings and application results obtained during the research process were formally documented and presented to the relevant stakeholders and the scientific community.

### Participants

#### Preschool teachers

The study sample consists of preschool children enrolled in preschool educational institutions in Cyprus during 2024–2025 academic year and the preschool teachers who work in these institutions.

In accordance with the needs analysis conducted at the planning stage of the action research, semi-structured interviews were conducted with 30 preschool teachers to explore their perspectives on phonological awareness skills. The demographic characteristics of the participating teachers are presented in Table 1.

**Table 1:** Demographic information of teachers participating in the action research needs analysis.

Variable	Sub-group	*n*	%
Professional seniority	1–5 years	15	50.0
	6–10 years	1	3.3
	11–15 years	5	16.6
	16–20 years	5	16.6
	21 years and above	4	13.3
Affiliation	Kindergarten	12	40.0
	Preschool	18	60.0
Phonological Awareness Training	Yes	7	23.3
	No	23	76.6

#### Preschool children

Prior to implementing the action plan, a convenience sampling strategy was employed within the needs analysis phase to observe activities targeting phonological awareness skills in preschool children. This sampling method aims to include in the research a readily accessible sample that can be reached efficiently (Creswell and Creswell, 2023). To this end, two classrooms comprising 35 children in a school chosen by the researcher, activities related to phonological awareness skills were observed. Information regarding the needs analysis observation group is presented in Table 2.

**Table 2:** Action research needs analysis observation group information.

Group	Age	Girls (*n*)	Girls (%)	Boys (*n*)	Boys (%)
Needs Analysis Observation Group	60 months	10	58.8	7	41.1
	60 months	9	50.0	9	50.0

The child participants chosen for the action plan to be implemented in the study were identified using a convenience sampling strategy, a non-probability sampling approach commonly utilized in qualitative research. This strategy facilitated the selection of an optimal host school where the study could be conducted in a smooth, effective, and uninterrupted manner. During the implementation phase of the action research, the action plan was implemented in a classroom comprising 17 children. Detailed characteristics of the intervention group are presented in Table 3.

**Table 3:** The action research implementation group information.

Group	Age	Girls (*n*)	Girls (%)	Boys (*n*)	Boys (%)
Implementation group	60 months	10	58.8	7	41.1

Although the intervention group initially consisted of 17 children, complete pre- and post-test data were available for 14 children. During the intervention period, two children withdrew from preschool, and one child with special educational needs was unable to complete the assessment protocol. Consequently, the quantitative analyses were conducted based on data from the remaining 14 children (*N* = 14). This sample was chosen in line with the contextual requirements of the action research and in accordance with the current number of students in the classroom where the researcher conducted the practice.

### Data collection instruments

In action research conducted in the field of education, it is considered important to carry out the implementation, observation, and problem-solving processes in a planned and systematic manner, to reflect on the results obtained, and to share them with researchers conducting similar studies (Mertler, 2020). It is observed that both qualitative and quantitative methods are used in the data collection process of action research. In determining data collection tools, it is important for the researcher to determine the frequency, time intervals, and specific techniques required to access data sources (Mills, 2018). In this research, both qualitative and quantitative methods were used in the data collection process. Observation enables for the examination of individuals’ behaviors, interactions, and learning processes in their natural environment (George, 2023). Conversely, interviews serve as an effective technique aimed at uncovering participants’ experiences, perceptions, and insights in depth (Mehrad, 2024).

### Semi-structured interview form

In the needs analysis phase of the research, interviews were conducted with 30 teachers using a semi-structured interview protocol developed by the researcher, and their perspectives on phonological awareness skills were gathered. The interview process spanned approximately 1 month. Expert guidance was sought in developing the activities aimed at developing phonological awareness skills within the framework of the identified needs. This study was conducted during the Spring semester of the 2024–2025 academic year. Each participant whose opinions were obtained was informed about the planned activities, resulting in the finalization of 36 activities over a two-week period. In the implementation phase of the action research, the children’s affective responses to the activities prepared to support phonological awareness skills were assessed using probing questions during activity implementation. After each activity, classroom observations and reflective notes were documented in research journals. In the study, the children’s phonological awareness post-intervention skill levels were re-assessed using the Early Literacy Test for Preschool Children (EROT).

### Early literacy test (EROT)

The Early Literacy Test (EROT), developed by Kargın et al. (2015) for children aged 60–72 months, is a standardized assessment tool with established validity and reliability. Having been developed as a standard evaluation tool, EROT was designed to evaluate early literacy skills. This test assesses early literacy skills in seven dimensions, namely Receptive Language Vocabulary, Expressive Language Vocabulary, General Naming, Functional Knowledge, Alphabetical Knowledge, Phonological Awareness, and Listening Comprehension. Whereas phonological awareness is evaluated through eight subtests in EROT, each of the remaining domains is assessed via a single subtest.

The content validity of EROT was established by an expert panel of faculty members representing four areas of expertise across three universities. The results showed that EROT has content validity. Construct validity was determined using exploratory and confirmatory factor analyses. A factor loading threshold of 0.32 was established, and items falling below this value were excluded from the subtests (Kargın et al., 2015).

Phonological awareness skills are evaluated using eight sub-tests. During administration, two practice items are presented using standardized directives. Children are allotted a 5-s response window per item. If a child provides an incorrect response within this period, the test administrator gives the correct answer. For actual test items, if a child responds incorrectly or fails to answer, the administrator proceeds to the next item neutrally without providing feedback. There are 16 example items and 23 question items in phonological awareness skills (Kargın et al., 2015).

Within the scope of the research, the cutoff point for the phonological awareness skills subtest was determined as 0.50, and the number of items corresponding to this cutoff point was determined as 16 (Kargın et al., 2015). In this respect, a score below 16 on the phonological awareness subtest indicates underdeveloped phonological awareness skills.

### Researcher observation forms and diaries

During the needs analysis process of the research, 35 children were observed. Within this scope, the activities implemented by the classroom teacher were monitored. Observations were made within the framework of phonological awareness skills, while the researcher maintained detailed field notes. In this process, the Early Literacy Test for Kindergarten Children (EROT) was administered to students in a designated classroom within the participating school to assess children’s baseline phonological awareness levels. Overall, this needs analysis phase spanned for approximately 1 month.

The developed activities were implemented over an 8-week period, 2 days a week, during the action research implementation phase.

### Action research application process

In accordance with the needs analysis results, expert guidance was sought during the development of activities targeting phonological awareness skills. The research was conducted during the Spring semester of the 2024–2025 academic year. Following a two-week evaluation process based on expert opinions, a total of 36 learning activities were finalized.

The developed activities were implemented over an 8-week period, 2 days a week, during the action research implementation phase. Academic journals, books, and web-based instructional resources were utilized in the preparation of the research activities (Schuele and Murphy, 2014; WEB1; Texas Education Agency, 2009; WEB 2; Pre-K Pages, n.d.; WEB 3; Reading Rockets, 1998; WEB 4; PreKinders, n.d.; WEB 5; Reading Resource, n.d.; WEB 6; Hand2Mind, n.d.; WEB 7; Reading BrightStart, n.d.; WEB. 8; PALS, n.d.; WEB. 9; Texas Primary Reading Inventory, n.d.).

When determining the implementation order of the activities, the phonological awareness training prepared by Parpucu and Dinç (2017), developed according to the structure of the Turkish language, and the SESFAR intervention program prepared by Akdal (2018) were taken into consideration. It is reported that children acquire syllable separation and rhyme awareness skills more easily due to the structural features of Turkish (Gökkuş and Akyol, 2020).

Action research process was conducted with an interrelated design consisting of planning, action, observation and reflection stages. At the planning stage, the educational needs of the participant children were determined and a structured series of phonological awareness activities was developed. It was aimed to structure the activities as mutually complementary, progressive, and repetitive teaching experiences to support the development of intended phonological awareness skills instead of being 36 independent interventions. It was not expected that developmental phonological awareness skills would be adequately and lastingly developed through a single instructional activity; therefore, opportunities were created for children to repeatedly practice the targeted skills across diverse content areas and contexts.

Weekly application order of the activities is as follows:

The weekly application order of the activities for developing the phonological awareness skills is as follows:

Week 1: Word syllable division skills (4 activities),

Week 2: Syllable blending skills (4 activities),

Week 3: Sentence segmentation skills (4 activities),

Week 4: Rhyme awareness skills (4 activities),

Week 5: Final sound matching (4 activities),

Week 6: Initial sound matching (4 activities),

Week 7: Final phoneme deletion (4 activities),

Week 8: Initial phoneme deletion (4 activities) and.

Phonemic segmentation skills (4 activities) were implemented.

In the implementation process, the researcher recorded her observations regarding the activities using observation logs. At the end of the application, EROT was implemented again so as to determine the phonological awareness skill levels of children again.

### The role of the researcher–practitioner

During this action research, the researcher also served as the classroom teacher and thus assumed the role of a teacher–researcher. For this reason, the researcher–teacher played an active role in planning the study, implementing the intervention, observing the children and reflecting on the application process. The action was carried out in the authentic classroom environment where the researcher worked. Pedagogical goals and targeted learning outcomes were maintained throughout the implementation, and to realize these outcomes 36 consecutive activities were implemented which were structured with a complementary, progressive and repetitive design. The researcher–teacher observed the participation, interest, reactions of children and the difficulties they experienced through observation logs and application records.

Although the overall action plan did not require substantial modification during implementation, the reflective process enabled the researcher–teacher to continuously evaluate children’s participation, engagement, responses, and difficulties. These reflections indicated that the planned activities were appropriate for the developmental level of the participating children and could therefore be continued as planned. Thus, reflection informed the pedagogical decision to maintain the overall action plan rather than substantially modify it.

This approach aligns with the teacher–researcher tradition which emphasizes that teachers can assume the role of a researcher in the process of researching and developing their own teaching practices (Schuele et al., 2007; Elliott, 2024).

### Analysis of the data

In qualitative research, data collection and data analysis processes are conducted concurrently. In this study, qualitative and quantitative analyses were integrated across the needs analysis, intervention design, and implementation phases of activity-centered direct instruction, and the determination of the research implementation process.

Quantitative analyses refer to showing the available data numerically and presenting the information in the form of frequency, percentage, mean, and standard deviation (Chand, 2025).

Qualitative content analysis is defined as an iterative and systematic technique which summarizes selected words from a text by grouping them into smaller parts according to predetermined codes and themes (Chand, 2025).

In the study, content analysis, a qualitative method, was used to analyze the interview transcripts obtained from teachers. In addition, quantitative techniques were used to compare pre-test and post-test scores in order to assess the level of phonological awareness skills in children prior to and following the action research intervention. Pre-test and post-test results are presented by calculating the arithmetic mean and standard deviation values. After the action research, observational logs maintained by the researcher were subjected to content analysis to generate qualitative findings supported by direct quotations.

### Validity and reliability analysis

It has been emphasized that reliability is more important than validity in qualitative studies, and that there are certain criteria (Lincoln and Guba, 1985). In this context, Lincoln and Guba (1985) proposed four criteria for trustworthiness: credibility, transferability, dependability, and confirmability. One or more of these four important criteria must be emphasized for the credibility of the obtained findings (Creswell and Creswell, 2023). For this reason, this research employed the reliability and validity studies defined in Table 4.

**Table 4:** Methods used for the reliability and validity of research.

Method	The process performed
Internal validity (credibility)	Using the triangulation method Participant confirmation Long-term interaction Giving direct quotes
External validity (transferability)	Using purposive sampling Describing the environment in detail Providing detailed information about the participants
Reliability (consistency)	Using the triangulation method Providing a detailed description of the research process Having another researcher examine the process and results
Objectivity (verifiability)	Reducing researcher bias Using the triangulation method

Including direct quotations in a qualitative study is a critical factor for the reliability and findings of the study. For this reason, the teachers were coded to keep confidential the personal information of the participant teachers, and their opinions were directly quoted.

- (Ö1): The first teacher whose opinions were sought.
- (Ö2): The second teacher whose opinions were sought, etc.

### Findings

In this section, findings obtained in line with the objectives of the research were presented according to the research questions. The findings are presented based on teacher opinions, children’s phonological awareness performance, and observation data acquired during the implementation process.

Findings regarding the first sub-objective of the study, “What are the preschool teachers’ perspectives on phonological awareness skills in the preschool period?” question, are given below.

### Preschool teachers’ definitions of the concept of phonological awareness

An analysis of teachers’ definitions of phonological awareness in Table 5 reveals that 67.7% of the definitions focused primarily on sound recognition and discrimination, followed by word and syllable awareness (25.8%), and awareness regarding the sound structure of language. Notably, only 3.2% of the definitions of teachers directly link phonological awareness to early literacy skills. This finding indicates that teachers’ conceptual understanding is predominantly confined to sound-based processes.

**Table 5:** Teachers’ views on the definition of the concept of phonological awareness.

Theme	*n*	%
Recognizing and distinguishing sounds	21	67.7
Word-syllable awareness	8	25.8
Sound structure of language	5	16.1
Rhyme awareness	4	12.9
Letter awareness	3	9.6
Early literacy acquisition	1	3.2

### Preschool teachers’ views on why phonological awareness skills are important for preschool children

Table 6 presents the teachers’ perspectives regarding the importance of phonological awareness skills. The findings indicate that 77.4% of teachers evaluate phonological awareness as a critical skill for literacy acquisition. Conversely, the proportion of teachers linking these skills to higher-level outcomes such as early literacy, academic success, and readiness for primary school was quite low at 3.2%.

**Table 6:** Teachers’ Views on the Importance of Phonological Awareness Skills.

Theme	*n*	%
Reading and writing	24	77.4
Language and speech	14	45.1
Alphabet and sound learning	6	19.3
Attention skills	2	6.4
Early literacy skills acquisition	2	6.4
Social development	1	3.2
Preparation for primary school	1	3.2
Academic success	1	3.2
Phonological awareness	1	3.2

### Preschool teachers’ views on activities they conduct to develop phonological awareness skills

When the activities regarding phonological awareness skills given in Table 7 are examined, it can be concluded that 35.4% of teachers use rhyme and poetry activities, whereas story reading activities have only 3.2% frequency. This, in turn, shows that activities are mostly based on rhythm and repetition.

**Table 7:** Activities aimed at developing phonological awareness skills.

Theme	*n*	%
Rhyme-poetry activities	11	35.4
Music-rhythm activities	7	22.5
Phonological awareness activities	7	22.5
Vowel recognition activities	6	19.3
Matching activities	5	16.1
Guessing-finding-signing activities	4	12.9
Attention-grabbing activities	2	6.4
Name writing activities	2	6.4
Imitation and listening activities	2	6.4
Spelling activities	2	6.4
Story-reading activities	1	3.2

## Methods and techniques used by preschool teachers in activities aimed at developing phonological awareness skills

Table 8 presents the instructional methods and techniques used by teachers, with game-based activities emerging as the most prevalent (29.0%), whereas strategies supporting cognitive structuring, such as concept mapping accounted for only 3.2 percent.

**Table 8:** Methods and techniques used in activities aimed at developing phonological awareness skills.

Theme	*n*	%
Play	9	29.0
Demonstration–performance	7	22.5
Drama	6	19.3
Listening to sounds-watching videos	6	19.3
Role playing-actualization	5	16.1
Observation	3	9.6
Question–answer	3	9.6
Brainstorming	2	6.4
Case study	2	6.4
Concept map	1	3.2

### Preschool teachers’ views on the materials they use in activities aimed at developing phonological awareness skills

As presented in Table 9, visual cards emerged as the most frequently utilized material (41.9%), whereas puzzles and cartoons accounted for the lowest proportion, at 3.2% each.

**Table 9:** Materials used in activities aimed at developing phonological awareness skills.

Theme	*n*	%
Cards	13	41.9
Technological devices	11	35.4
Real and natural objects	8	25.8
Musical instruments	6	19.3
Toys and puppets	5	16.1
Letter cards–alphabet	4	12.9
Sound cards	4	12.9
Playdough	4	12.9
Books–worksheets	4	12.9
Cartoons	1	3.2
Puzzles	1	3.2

## Evaluation approaches and methods used by preschool teachers at the end of activities related to phonological awareness skills

Table 10 demonstrates that, in terms of evaluation approaches, question–answer leads the list with 54.8%, followed by observation methods with 29%, whereas project evaluation, preparation of progress reports, discussion, and process evaluation remained limited at 3.2 percent.

**Table 10:** Assessment approaches and methods used in activities aimed at developing phonological awareness skills.

Theme	*n*	%
Question–answer	17	54.8
Observation	9	29.0
Listening–conversation	6	19.3
Project evaluation	1	3.2
Preparation of progress report	1	3.2
Discussion	1	3.2
Process evaluation	1	3.2

### Preschool teachers’ views on suggested training activities for developing phonological awareness skills

Table 11 reveals that among teachers’ recommendations regarding phonological awareness skills, audio-visual activities emerged as the most frequent category (29.0%), followed by game-based activities (25.8%), whereas imitation-exploratory, listening, nature-based, and technology-assisted activities accounted for the lowest proportions, at 3.2% each.

**Table 11:** Suggested activities for developing phonological awareness skills.

Theme	*n*	%
Audio-visual activities	9	29.0
Games	8	25.8
Rhyme-singing activities	5	16.1
Name recognition activities	3	9.6
Creating a classroom corner	2	6.4
Drama activities	2	6.4
Sorting-classification activities	2	6.4
Music activities	2	6.4
Imitation-discovery activities	1	3.2
Listening activities	1	3.2
Natural environment activities	1	3.2
Technological activities	1	3.2
No suggestions	3	9.6

### Suggestions for methods and techniques that can be used in phonological awareness training for preschool teachers

Table 12 presents teachers’ recommendations concerning instructional methods and techniques for phonological awareness education. Game-based activities (25.8%) and practical exercises (19.3%) emerged as the two most frequently recommended approaches. Conversely, brainstorming and concept mapping represented the least frequently suggested strategies, at 3.2% each.

**Table 12:** Suggested methods and techniques for phonological awareness training.

Theme	*n*	%
Game	8	25.8
Application-exercise	6	19.3
Question–answer	3	9.6
Discussion	3	9.6
Simulation	2	6.4
Observation	2	6.4
Learning by doing	2	6.4
Brainstorming	1	3.2
Concept map	1	3.2
No suggestions	2	6.4

### Suggestions for materials that can be used in phonological awareness training for preschool teachers

Table 13 examines the material suggestions of teachers for use in phonological awareness training. It was concluded that audio-visual materials have the highest frequency at 61.2% whereas musical instruments have the lowest with 6.4%.

**Table 13:** Suggestions for materials that can be used in phonological awareness training.

Theme	*n*	%
Audiovisual-tactile materials	19	61.2
Technological materials	8	25.8
Books	4	12.9
Natural materials	3	9.6
Worksheets–applications	3	9.6
Musical instruments	2	6.4
No suggestions	1	3.2

### Suggested assessment approaches and methods for use in phonological awareness training for preschool teachers

Table 14 presents the findings regarding evaluation approach and method suggestions of teachers that could be used in phonological awareness education. According to the table, observation emerged as the most recommended approach (38.7%), followed by performance-based assessment (29.0%). In contrast, progress reporting and project-based assignments represented the least frequently suggested methods, accounting for only 3.2% each.

**Table 14:** Suggested assessment approaches and methods for phonological awareness training.

Theme	*n*	%
Observation	12	38.7
Performance evaluation	9	29.0
Question–answer	7	22.5
Preparation of progress report	1	3.2
Project-based assignment	1	3.2
No suggestions	2	6.4

Findings regarding “What are the baseline phonological awareness skill levels of preschool children aged 48–60 months?”, which is the second sub-objective of the study, are given in Table 15.

**Table 15:** Action research participant group’s pre-test scores on phonological awareness skills.

Phonological awareness skill performances		Rhyme awareness	Sentence segmentation	Syllable segmentation	Syllable blending	Initial sound matching	Final sound matching	Initial phoneme deletion	Final phoneme deletion	Total score
Participants	P1	2	0	0	0	1	2	0	0	5
	P2	4	1	2	3	1	2	0	0	13
	P3	4	3	0	4	3	3	0	0	17
	P4	3	0	0	0	3	2	0	0	8
	P5	2	0	0	3	1	2	0	0	8
	P6	1	1	0	3	2	1	0	0	9
	P7	4	0	0	3	2	2	0	0	11
	P8	1	0	0	1	1	0	0	0	3
	P9	1	0	0	0	3	2	0	0	6
	P10	3	0	0	4	2	3	0	0	12
	P11	2	0	4	4	3	3	0	0	16
	P12	2	0	2	4	2	2	0	0	14
	P13	4	0	0	0	1	3	0	0	8
	P14	3	0	0	0	4	1	0	0	8

### Levels of phonological awareness skills in preschool children aged 48–60 months

Table 15 presents the baseline evaluation of children’s phonological awareness skills measured via EROT. EROT scores are examined in terms of rhyme awareness, sentence segmentation, syllable segmentation, syllable blending, initial sound matching, final sound matching, initial phoneme deletion, and final phoneme deletion skills. The potential score range for each subtest spans from 0 to 4. Total baseline scores among participants ranged from 3 to 17. Findings show that children exhibited lower performance in phonemic awareness and syllable-level tasks. Notably, the majority of the intervention group scored below the established cutoff threshold.

Findings regarding the third sub-objective of the study, “What kind of action plan should be developed to improve phonological awareness skills?” are given in Tables 16, 17.

**Table 16:** Activity observation list.

Activity	Application period (min)	Number of students (*n*)
Spring flower picking	30	16
Arda who loves kites	40	16
Pink	30	16
The Sound ‘Ü’ and riddles	40	15
A day at the library	30	16
Name awareness	40	16
Opposite concepts	30	15
Synonyms	40	15

**Table 17:** Analysis of observed activities related to phonological awareness skills.

Activity	Rhyme awareness	Word awareness	Syllable awareness	Phonological awareness
Spring flower picking	_	+	_	+
Arda who loves kites	_	+	_	+
Pink	_	+	_	_
The Sound ‘Ü’ and riddles	_	+	_	+
A day at the library	_	_	_	_
Name awareness	_	_	+	+
Opposite concepts	_	+	_	_
Synonyms	_	+	_	_

+ Skill addressed in activity; − Skill not addressed in the activity.

### Findings regarding action research aimed at developing phonological awareness skills

As summarized in Table 16, eight instructional sessions were observed over an eight-week period, conducted at a frequency of one activity per week. The duration of each activity ranged from 30 to 40 min. Within the 17-child classroom, one child with special educational needs was absent from all sessions, whereas all other children participated consistently across all implementation sessions.

An analysis of the eight instructional activities presented in Table 17 revealed that six targeted word awareness, four targeted phonemic awareness, and one targeted syllable awareness. Notably, activities addressing rhyming skills—a foundational component of phonological awareness—were entirely absent among the observed practices.

Observations made within the scope of needs analysis show that phonological awareness in preschool settings is not addressed in a systematic and balanced manner. It has been determined that the observed activities mostly focus on word awareness and that activities regarding rhyme awareness are absent.

Findings regarding the question “What are the phonological awareness skill levels of children prior to and following the action research implementation?”, which is the fourth sub-objective of the research, are given in Tables 18–20.

**Table 18:** Action research participant group phonological awareness skills pre-test scores.

Phonological awareness skills	*n*	*M*	*SD.*	Min.	Max.
Rhyme awareness	14	2.57	1.15	0.00	4.00
Word separation	14	0.35	0.84	0.00	4.00
Syllable separation	14	0.57	1.22	0.00	4.00
Syllable blending	14	2.07	1.77	0.00	4.00
Initial sound matching	14	2.07	0.99	0.00	4.00
Final sound matching	14	2	0.87	0.00	4.00
Initial phoneme deletion	14	0	0	0.00	4.00
Final phoneme deletion	14	0	0	0.00	4.00
Total phonological awareness	14	9.85	4.12	0.00	4.00

**Table 19:** Phonological awareness skills post-test score of the action research participant group.

Phonological awareness skill performances		Rhyme awareness	Sentence segmentation	Syllable segmentation	Combining syllables	Initial sound matching	Final sound matching	Initial phoneme deletion	Final phoneme deletion	Total score
Action research participant group	P1	4	2	4	4	4	4	1	3	26
	P2	4	4	4	4	4	3	1	3	27
	P3	4	3	4	4	4	4	1	4	28
	P4	4	2	4	4	4	4	1	3	26
	P5	4	2	4	4	4	4	2	1	25
	P6	4	4	4	4	4	4	1	3	28
	P7	4	3	4	4	4	3	1	2	25
	P8	4	2	2	4	4	4	0	2	22
	P9	3	4	4	4	4	3	1	2	25
	P10	4	1	4	4	4	4	2	1	24
	P11	4	4	4	4	4	4	1	2	27
	P12	4	3	4	4	4	3	1	2	25
	P13	4	3	4	4	4	3	1	2	25
	P14	4	4	4	4	4	3	2	3	28

**Table 20:** Phonological awareness skills post-test mean scores.

Phonological awareness skills	*n*	*Mean*	*SD.*	*Min.*	*Max.*
Rhyme awareness	14	3.32	0.26	0.00	4.00
Word separation	14	2.92	0.99	0.00	4.00
Syllable separation	14	3.85	0.53	0.00	4.00
Syllable blending	14	4.00	0	0.00	4.00
Initial sound matching	14	4.00	0	0.00	4.00
Final sound matching	14	3.57	0.51	0.00	4.00
Initial phoneme deletion	14	1.14	0.53	0.00	4.00
Final phoneme deletion	14	2.35	0.84	0.00	4.00
Total phonological awareness	14	25.78	1.71	0.00	4.00

Table 18 shows that the children’s pre-test mean scores were below the cutoff score of 16 in all sub-dimensions of phonological awareness skills, with an average score of 9.85.

Table 19 presents the results of the Early Literacy Test (EROT), used to assess the phonological awareness skill levels of children in the Action Research Participant Group after the action research.

As summarized in Table 19, post-test EROT scores were evaluated across rhyme awareness, sentence segmentation, syllable segmentation, syllable blending, initial sound matching, final sound matching, initial phoneme deletion, and final phoneme deletion. The potential score range for each subtest spans from 0 to 4. Total post-test scores among participants ranged from 22 to 28. All participants in the intervention group scored above the established cutoff threshold of 16.

Table 20 presents the mean post-test scores of the children comprising the participant group.

When the post-test scores of children after the action research given in Table 20 are examined, a remarkable increase is observed in all sub-dimensions. It is particularly noteworthy that full scores were achieved in Syllable blending and initial sound matching skills. It has been determined that post-test mean score is 25.78, which is significantly above the cut-off score of 16.

### The researcher’s observations regarding the implementation of activities targeting phonological awareness skills

Findings regarding the question “What are the researcher’s observations regarding the implementation of activities designed to target phonological awareness skills?”, which is the fifth sub-objective of the research, are given in Table 21.

**Table 21:** Researcher observations on activities developed based on phonological awareness skills.

Sequence and title of the activities	Scope of skill	Class participation in the activity	Interested in the activity	Liked the activity
1. Spell the words	Syllable awareness	X	X	X
2. How many syllables are there?				
3. Find the missing syllable				
4. Say the remaining syllable				
1. Combine and say	Syllable awareness	X	X	X
2. Find the word				
3. How many paper sheets did you use?				
4. Color according to syllable count				
1. Complete the sentence	Word awareness	X	X	X
2. Word order in the sentence				
3. Incorrect word				
4. Number of words in the sentence				
1. Underline the word with a similar pronunciation.	Rhyme awareness	X	X	X
2. Match the words with similar pronunciations.				
3. Delete the word that does not rhyme.				
4. Find the deleted rhyming word.				
1. Identify the last sound of the word	Phonological awareness	X	X	X
2. Match the words according to their last sounds				
3. Find the word with a different last sound				
4. Find the words with the same last sounds				
1. Identify the first sound of the word	Phonological awareness	X	X	X
2. Match the words according to their first sounds				
3. Place words with the same first sound in the basket				
4. Find the word that begins with a different sound				
1. Delete sound	Phonological awareness	X	X	X
2. Say the new word				
3. What’s left?				
4. Delete and match				
1. Delete sound	Phonological awareness	X	X	X
2. Say the new word				
3. Delete and match				
4. What’s left?				
1. Break down the words into their sound units.	Phonological awareness	X	X	X
2. How many sounds are there?				
3. Let us combine the sounds.				
4. Find the word				

X demonstrates the existence of observation.

Table 21 presents the researcher observations regarding the 36 activities developed. The findings demonstrate that the activities were found to be enjoyable by the children, that participation was full at every activity, which made the learning process more entertaining and exploratory.

To contextualize and complement the quantitative findings presented in Table 21, reflective journals maintained by the researcher–practitioner throughout the eight-week implementation were analyzed. These reflective logs yielded contextual insights into children’s engagement, skill acquisition trajectories, activity feasibility, and pedagogical challenges. Content analysis of these logs yielded four main themes: (1) student engagement and motivation, (2) transfer and progression of targeted skills, (3) feasibility of activity implementation, and (4) areas of relative difficulty.

“The visual materials I utilized in the first week’s activities focusing on syllable awareness highly engaged the children. After grasping the skill, the children began playing games by associating the names of objects in the classroom and the schoolyard with the activities. The activity was executed seamlessly within the scheduled timeframe.”

“In the second week of activities focusing on syllable awareness, the children were able to complete the new activities more easily because they retained the syllable concepts acquired in the previous week. In particular, we incorporated a name-based game into the syllable-counting activity. The duration and procedural steps of the activity proceeded according to plan.”

“In the first two word awareness activities, I used words the children were already familiar with, so their engagement in word-focused activities was notably high. In subsequent activities, exposure to unfamiliar words piqued their curiosity. I was able to complete all implementation phases within the allocated timeframe.”

“Rhyme awareness activities were among the most enjoyable for the children. After noticing similarities in the endings of words, they began to spontaneously generate rhyming words with their peers. Throughout the activity, the children were observed to be having fun and actively participating. “The duration of the activity, the materials and the stages of its implementation were done as planned.”

“In activities targeting phonetic awareness it was observed that the children performed easier matching activities based on the first and last sounds. The visuals in the materials I used were particularly appealing to the children. The activities of initial phoneme deletion, final phoneme deletion, and phoneme segmentation were not as easy for the children as the other activities, but they still attracted their interest and their participation continued.”

“I found the duration of the activities appropriate in terms of the children’s developmental levels. The children maintained sustained attention without showing signs of fatigue or disinterest and enjoyed themselves.”

“During the implementation process, a brief evaluation was conducted with observing classroom teacher. The teacher reported that the children participated willingly in the activities and showed a high level of interest in the activities. These observations were consistent with the researcher–practitioner’s reflections recorded throughout the implementation.”

An analysis of the researcher–practitioner’s reflective logs indicates that systematically planned and executed phonological awareness-based practical activities support the acquisition of these target skills in a participatory classroom environment.

## Discussion

The findings of this study are discussed in light of the extant literature, focusing on the current state of phonological awareness skills in preschool children, teacher practices, and the changes observed following the implementation of the developed activities. Findings show that preschool teachers have a limited conceptual understanding of phonological awareness, which is subsequently reflected in their instructional practices. The vast majority of teachers define phonological awareness as the ability to recognize and distinguish sounds, which indicates that the multifaceted nature of this skill, encompassing subcomponents such as rhyme, syllable, word, and phoneme awareness, is not sufficiently understood. This finding underscores the imperative to strengthen phonological awareness content knowledge in teacher education and in-service training processes (Veríssimo et al., 2021; Choi et al., 2024).

Teachers primarily associated the significance of phonological awareness with early literacy acquisition, which shows that they are aware of its role in early literacy processes. Indeed, it is frequently emphasized in the literature that early development of phonological awareness skills strongly predicts children’s reading success and reading comprehension skills in later years (Yeh and Connell, 2008; Ertekin and Çakmak, 2024; Perišić and Minić, 2025). However, observational data suggest that this theoretical awareness fails to translate into systematic, balanced instructional practices.

Research findings show that teachers often incorporate rhymes, poems, and game-based activities into their phonological awareness programs, which coincides with studies highlighting the importance of learning through play in the preschool period (McLachlan and Arrow, 2014; Arslan Çiftçi et al., 2025). Furthermore, it is seen that activities are mostly limited to rhyme awareness and that there are few applications that comprehensively address syllables, words, and particularly phonemic awareness. However, addressing the sub-dimensions of phonological awareness skills as a whole is a critical prerequisite for successfully transitioning children into formal reading instruction (Gillon, 2002; Hsin, 2017; Veríssimo et al., 2021).

Similarly, teachers’ heavy reliance on traditional question-and-answer techniques provides a limited scope for assessing a developmental and process-oriented skill such as phonological awareness. In the literature it is recommended that the evaluation of these skills involves using a combination of observation, performance-based assessment, and multiple measurement approaches (Callaghan et al., 2016; Parpucu and Güler Yıldız, 2023). This finding underscores the critical need for professional development regarding authentic assessment approaches.

Findings obtained regarding the phonological awareness levels of the children indicated that the majority of children were identified as at-risk prior to the action research. Pre-test scores were substantially below the established EROT cutoff threshold, which shows that these skills are not adequately supported during the preschool years. This outcome aligns with prior studies indicating that children with weak phonological awareness skills may experience challenges in subsequent reading acquisition and overall academic performance (Maslanka and Joseph, 2002; Özen Altınkaynak, 2019; Cabell et al., 2021).

The findings acquired within the scope of needs analysis indicate that teachers conducted activities regarding word awareness, phoneme awareness, and syllable awareness, but that no activity was performed on rhyme awareness. Bryant et al. (1990) posited that rhyme awareness serves as a foundational precursor in the acquisition of phonological awareness skills. In addition, it is stated that by attending to recurring sound patterns across words and ensuring these letters are in the same order, children develop an awareness that contributes to the development of their reading and writing skills (Cabell et al., 2021). Several studies demonstrate that the acquisition of phonological skills is highly effective in reading success (Cabell et al., 2021; Choi et al., 2024; Perišić and Minić, 2025). Consequently, teachers play a crucial role in helping preschool children acquire this skill, which is of paramount importance for their subsequent academic development. Extant research underscores that the efficacy of early literacy instruction hinges upon having appropriate curricula and competent teachers to implement them (Pullen and Justice, 2003; Justice et al., 2017).

An increase in phonological awareness scores was observed, with the mean EROT score increasing from 9.85 at pre-test to 25.78 at post-test. This within-group change indicates higher phonological awareness scores following the implementation period; however, given the action research design and the absence of a control group, these changes cannot be attributed solely to the implemented activities. This finding is consistent with prior empirical studies reporting positive outcomes associated with phonological awareness instruction (Turan and Akoğlu, 2011; Carson et al., 2013; Parpucu and Güler Yıldız, 2023).

Various developmental levels between subdimensions can be evaluated considering the initial skill levels of children and the cognitive functions required by phonological awareness tasks. The high post-test mean scores achieved in syllable blending and initial sound matching skills (*M* = 4.00) show that children can perform the tasks associated with these skills more easily. In contrast, the post-test and pre-test mean scores for initial phoneme deletion skill were 1.14 and 0.00, respectively, which shows that this was a very limited skill at the beginning and even after the implementation, it did not reach as high a level as some of the other sub-skills. The literature shows that phonological awareness skills of children follow a developmental order (Anthony et al., 2003; Goodrich and Lonigan, 2014). At first children are sensitive to words and syllables, which are larger and more concrete sound units, and then progress towards smaller and more abstract sound units such as phonemes; they can generally combine sounds before segmenting words into their component sounds. This developmental process shows that sound deletion skill develops after other phonological awareness skills (Anthony et al., 2003; Goodrich and Lonigan, 2014; Walley et al., 2003). In this context, it is suggested that tasks requiring more advanced phonological manipulation-such as phoneme deletion-may be developmentally linked to higher-level skills; specifically, those requiring children not only to perceive the sound structure of a word but also to mentally remove a specific phoneme and process the new word formed by the remaining sounds (de Graaff et al., 2011; Schuele and Murphy, 2014). For this reason, the relatively low performance in initial phoneme deletion should not be seen as the inadequacy of the intervention; rather, it should be interpreted with caution taking into consideration the children’s initial level and the level of phonological processing required by the task. Another critical factor to consider is the frequency of words. The literature associates the phoneme deletion performance of children with the frequency with which a word appears in speech or text. It is reported that words with higher frequency are more encountered compared to words with lower frequency and that they overlap more phonologically, as a result of which words with higher frequency have more opportunity to undergo further lexical restructuring, such as phoneme loss, compared to words with lower frequency (Hogan et al., 2011; Goodrich and Lonigan, 2014). In this context, such experiences were more limited for words encountered less frequently by the children included in this study, which may have hindered the execution of processes requiring lexical restructuring, such as the deletion of the initial sound of a word. Yücesan-Durgunoğlu and Öney (1999) reached similar conclusions and found that Turkish-speaking children performed better on the task of deleting the final phonemes than on the task of deleting the initial phonemes.

The research found that final phoneme deletion performance is higher than that of initial phoneme deletion. The relatively lower performance in initial phoneme deletion is thought to be related to the morphological structure of Turkish language. In agglutinative languages like Turkish (Mete and Ceylan, 2015), the systematic attachment of suffixes to the end of words can allow children to encounter the word-final phonemes—and the changing functions of these sounds within word structures—more frequently. This, in turn, helps children to gain more experience in identifying and deleting the final phonemes from words and thus exhibit higher performance in the final phoneme deletion task.

Observational evidence and reflective logs indicated high levels of children’s engagement and interest throughout the implementation, while the quantitative findings showed higher phonological awareness scores at post-test. These results are consistent with studies highlighting that children exposed to phonological awareness instruction during the preschool period can acquire foundational reading prerequisites more robustly (Ong, 2008; Li et al., 2025).

### Limitations

A primary limitation of this study is that it was conducted within a single early childhood center and with a limited number of children, thereby constraining the generalizability of the findings to different socio-economic, cultural and educational contexts. Future studies in different educational settings and with larger samples would contribute to expanding and validating these findings and assessing the applicability of the proposed intervention across diverse settings.

In accordance with the context-bound nature of action research, the sample was selected purposefully based on localized implementation requirements rather than to achieve statistical representativeness. Consequently, the quantitative findings are interpreted strictly as evidence of intra-group progress among the participating children, rather than as generalizable claims to the broader preschool population.

Second, the effects of the intervention program are evaluated immediately following the eight-week implementation period. For this reason, inferences regarding the long-term sustainability of developmental changes observed in children are limited. Conducting longitudinal studies which cover the transition process to elementary school can allow for more comprehensive evaluation of the impact of these foundational gains on subsequent reading development (Lonigan et al., 2008; National Early Literacy Panel, 2008).

Another critical limitation of the study is that the intervention was conducted without a control group, which necessitates caution when attributing observed gains solely to observed in children solely to the intervention program. However, the study was designed as action research focused on the development, application and evaluation of an intervention process based on an educational need identified in a natural preschool environment rather than an experimental impact comparison. For this reason, the objective was not a comparative impact analysis but an examination of the applicability of developed activities, their contribution to the learning processes of children, and their functioning in classroom practices (Kemmis et al., 2014; Stringer, 2020).

Finally, the researcher navigated the dual role of researcher–practitioner, which introduces potential researcher bias during data interpretation process. To mitigate this limitation, methodological triangulation was employed by synthesizing data obtained from interviews, observations, researcher logs, and quantitative evaluations (Herr and Anderson, 2015).

The implementation process was followed through the observation logs and implementation records of the researcher; however, an independent implementation fidelity checklist was not used. Therefore, the consistency of the intervention was not independently verified, which is another limitation of the study.

### Implications for practice and future research

The findings of this study suggest that phonological awareness in the preschool period may benefit from systematic and developmentally appropriate instructional support. Prior to the study, the majority of children were classified as at-risk in terms of phonological awareness and there were notable gaps in teachers’ pedagogical content knowledge and instructional execution in this area, which implies that early literacy applications are not systematically supported in preschool settings. Following the implementation of structured activities tailored to practitioner feedback, needs analysis findings, and the developmental characteristics of the children, higher post-test phonological awareness scores were observed among the participating children. These findings highlight the practical value of planned, developmentally sequenced, and systematically structured activities for supporting phonological awareness skills in preschool classroom settings.

Based on research findings, it is recommended that phonological awareness instruction be integrated into preschool education programs in a more systematic and developmentally structured manner. Specifically, rhyme awareness, syllable awareness, word awareness, and phoneme awareness skills should be addressed within a complementary developmental process, which can help children start primary school with a stronger foundation in literacy. Furthermore, it is essential that preschool teachers be supported through pre-service and in-service training for the pedagogical content knowledge, assessment literacy, and instructional delivery regarding phonological awareness skills. Empirical evidence suggests that teacher competence and the learning experiences offered to children may play an important role in supporting early literacy skills.

It is recommended that future research replicate and expand similar intervention models across diverse geographical regions, varied age brackets, and larger sample sizes. In addition, conducting longitudinal studies examining the impact of the foundational skills acquired during this intervention on reading accuracy, reading fluency and reading-comprehension skills at elementary school level can make essential contributions to the field. It is suggested that multi-dimensional studies examining the complex interplay between teacher competencies, classroom practices, and children’s early literacy development should increase. Demonstrating the long-term effects of high-quality phonological awareness interventions in early childhood would significantly advance both theoretical frameworks and evidence-based practices in the field of early literacy.

## Conclusion

Taken together, these findings suggest that phonological awareness during the preschool period may benefit from systematic instructional support rather than being left solely to children’s natural developmental processes. Prior to the research, teachers demonstrated notable gaps in pedagogical content knowledge and instructional execution in this area, and the majority of children were classified as at-risk. These findings highlight the importance of systematic support in early literacy instructional practices.

However, following the implementation of structured activities tailored to educator perspectives, needs analysis data, and children’s developmental profiles, substantial score gains were recorded across children’s phonological awareness measures. These findings suggest that structured, developmentally sequenced instructional practices may support phonological awareness skills, while acknowledging that, in the absence of a control group, these observed changes cannot be attributed exclusively to the implemented activities. In this regard, the study not only reaffirms the importance of phonological awareness skills, but also provides empirical and practical insights into how a developmentally structured instructional process can be implemented within preschool settings while actively addressing practitioner needs.

## Data Availability

The raw data supporting the conclusions of this article will be made available by the authors, without undue reservation.
